# Design of a 60.8 K superconducting hydride LiMgZr_2_H_12_ at ambient pressure *via* lithium substitutional doping

**DOI:** 10.1039/d6ra02577c

**Published:** 2026-05-29

**Authors:** Qun Wei, Xinyu Wang, Jing Luo, Meiguang Zhang, Bing Wei

**Affiliations:** a School of Physics, Xidian University Xi'an 710071 China qunwei@xidian.edu.cn bwei@xidian.edu.cn; b College of Physics and Optoelectronic Technology, Baoji University of Arts and Sciences Baoji 721016 China zhmgbj@126.com

## Abstract

High-pressure hydrogen-rich compounds have long been regarded as promising room-temperature superconductor candidates; however, their practical applications are limited by their reliance on extreme compression. This study explores hydrogen-rich superconductors that may be stable at ambient pressures. Inspired by recent investigations of the MgZrH_2*n*_ family, the LiMgZr_2_H_12_ structure with a *Pmmm* symmetry was constructed, and its thermodynamic, mechanical, and dynamical stability were evaluated using first-principles calculations. Electron–phonon coupling (EPC) analysis suggests that LiMgZr_2_H_12_ reaches a superconducting critical temperature (*T*_c_) of 60.8 K at ambient pressure. Compared with MgZrH_6_, the introduction of Li atoms significantly increases the contribution of hydrogen atoms to the electron density of states near the Fermi level and enhances the EPC constant (*λ*) of the LiMgZr_2_H_12_ structure. LiMgZr_2_H_12_ exhibits a superconducting figure of merit of 1.56, which is significantly greater than that of MgZrH_6_, demonstrating its outstanding potential for practical applications. This work guides ambient-pressure design of high-*T*_c_ hydrides.

## Abstract 2

Inspired by MgZrH_6_, we designed the quaternary superhydride LiMgZr_2_H_12_ EPC calculations show that it is dynamically stable at ambient pressure and exhibits a superconducting *T*_c_ of 60.8 K.

## Graphical abstract

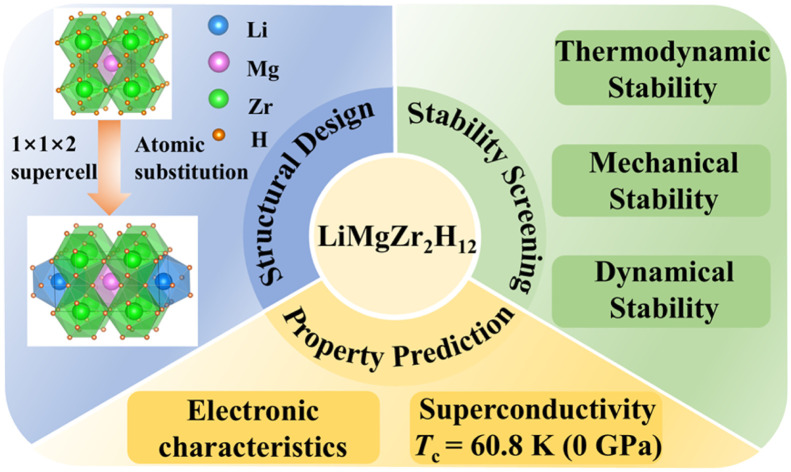

## 1. Introduction

Superconductors have long attracted significant interest for their broad application potential, and the pursuit of high-temperature or even room-temperature superconductivity remains a key research focus in condensed matter physics and materials science. Over the past decade, breakthroughs have been achieved in studies on hydrogen-rich superconductors. Binary hydrides including H_3_S,^1^ CaH_6_,^2^ LaH_10_,^3^ YH_9_,^4^ and YH_6_ (ref. 5) exceed the conventional low-temperature superconductivity regime, achieving superconducting critical temperatures (*T*_c_) above 200 K. The successful theoretical and experimental identification of these binary hydrides has further accelerated the development of high-pressure hydrides. Remarkably, several ternary hydrides predicted in recent years, such as Li_2_MgH_16_,^6^ Li_2_NaH_17_,^7^ Li_2_Na_3_H_23_,^7^ and LaSc_2_H_24_,^8^ are theoretically expected to exhibit high *T*_c_ values approaching or even exceeding room temperature under high pressures. However, most hydride superconductors are stable only above 150 GPa, severely limiting practical applications, making the realization of high-temperature superconductivity at low or ambient pressure a critical challenge.

Doping is one of the most effective strategies for tuning material properties and has been extensively demonstrated in multicomponent superconducting hydride systems.^9,10^ For example, Bi *et al.*^11^ introduced equiatomic La–Ce substitution into the clathrate framework of CeH_9_; the resulting ternary (La,Ce)H_9_ alloy exhibits *T*_c_ values of 148–178 K between 97 and 172 GPa, representing an enhancement of approximately 80% compared with binary CeH_9_. The LaBeH_8_ structure synthesized by introducing Be into the La–H framework exhibits a critical temperature *T*_c_ ≈ 110 K, as measured by electrical transport at approximately 80 GPa.^12^ Similarly, the introduction of a small amount of Al into the La–H system *via* interstitial doping effectively reduces the stabilization pressure and enhances superconducting performance. With an appropriate Al content, the hexagonal *P*6_3_/*mmc*-LaH_10_ phase, which is originally stable only at ultra-high pressures, can be stabilized at approximately 146–183 GPa, and a maximum *T*_c_ of 223 K is achieved at 164 GPa.^13^ Both theoretical and experimental studies have demonstrated that, in superhydride systems, elemental doping can substantially reduce the pressure required for structural stabilization and increase the *T*_c_. For multicomponent superhydrides, such as quaternary or quinary systems, constructing a complete phase diagram is highly challenging. Consequently, substitutional doping of preexisting hydrides that already exhibit excellent superconducting properties is a more efficient strategy than performing computationally expensive full-space exhaustive searches.

Among the various candidates proposed for reducing the required pressure, the Mg–Zr–H system composed of the light element Mg and the transition metal Zr has attracted our attention. Within the MgZrH_2*n*_ series, the *Pm*3̄ -MgZrH_6_ phase is estimated, based on the Gor'kov–Kresin equation, to exhibit a *T*_c_ value of 80.3 K at 36 GPa and a superconducting figure of merit (*S*) of 1.51, demonstrating the excellent superconducting potential.^14^ Motivated by the excellent superconducting performance of MgZrH_6_, we propose constructing a supercell and introducing the light element Li to reduce the pressure required for structural stabilization, thereby further enhance its superconducting properties. On the one hand, the atomic radius of Li is comparable to that of Mg, whereas its lower electronegativity allows for greater charge transfer from Li to H. On the other hand, the low atomic mass of Li can reduce the effective lattice mass and increase the logarithmic average phonon frequency *ω*_log_. Accordingly, we construct a 1 × 1 × 2 MgZrH_6_ supercell along the lattice directions to obtain a Mg_2_Zr_2_H_12_ supercell and then substitute one Mg atom with Li, ultimately yielding a LiMgZr_2_H_12_ quaternary hydride. In this study, we evaluate the thermodynamic, mechanical, and dynamical stability of the LiMgZr_2_H_12_ structure and systematically evaluate its superconducting properties *via* electron–phonon coupling (EPC) calculations. Our study provides useful guidance for future theoretical and experimental explorations of ambient-pressure high-temperature superconductors.

## 2. Computational details

Geometry optimizations and related property calculations for LiMgZr_2_H_12_ were performed within the framework of density functional theory (DFT) using the Perdew–Burke–Ernzerhof (PBE)^14–17^ parametrization of the generalized gradient approximation (GGA),^18^ as implemented in the Vienna *Ab initio* Simulation Package (VASP).^19^ The electron–ion interaction is described by projector augmented wave potential.^20^ A plane-wave cutoff energy of 600 eV was employed, and the Monkhorst–Pack *k*-grid^21^ with a spacing of 2π × 0.02 Å^−1^ was used to ensure adequate convergence. Structural relaxations were performed until the total energy and residual atomic forces converged to within 1 × 10^−5^ eV per atom and 1 × 10^−3^ eV Å^−1^, respectively. The single-crystal elastic constants were obtained by fitting linear stress–strain relations.^22^ Dynamic stability was evaluated using the finite displacement approach, and phonon spectra were calculated using the PHONOPY package.^23^ The crystal orbital Hamiltonian population (COHP) and the corresponding integrated COHP (ICOHP) values were obtained using the LOBSTER code.^24,25^ EPC was calculated using the Quantum ESPRESSO package^26^ with a plane-wave kinetic energy cutoff of 80 Ry. For Brillouin-zone sampling, a 24 × 24 × 12 *k*-point mesh together with a Gaussian smearing of 0.05 Ry was adopted to achieve convergence, while a 6 × 6 × 3 *q*-point mesh was employed to compute the EPC constant. The superconducting critical temperature was estimated using the Allen–Dynes modified McMillan equation.^27^

## 3. Results

**Fig. 1:**
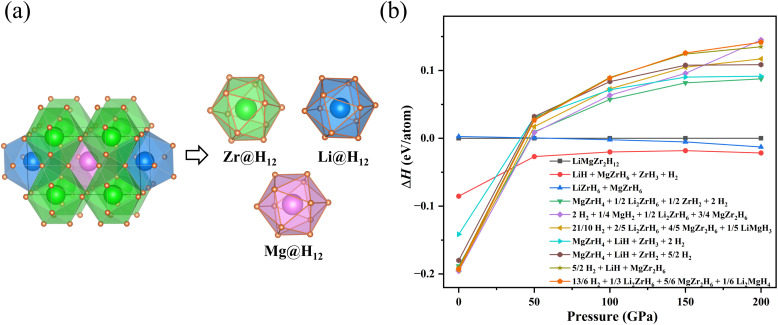
(a) Crystal structure of the quaternary hydride LiMgZr_2_H_12_ with *Pmmm* symmetry. (b) Relative enthalpies (Δ*H*) of the *Pmmm* LiMgZr_2_H_12_ with respect to decomposition into the listed ternary, binary hydrides and the elemental phases mentioned in previous literature from 0 to 200 GPa.

**Fig. 2:**
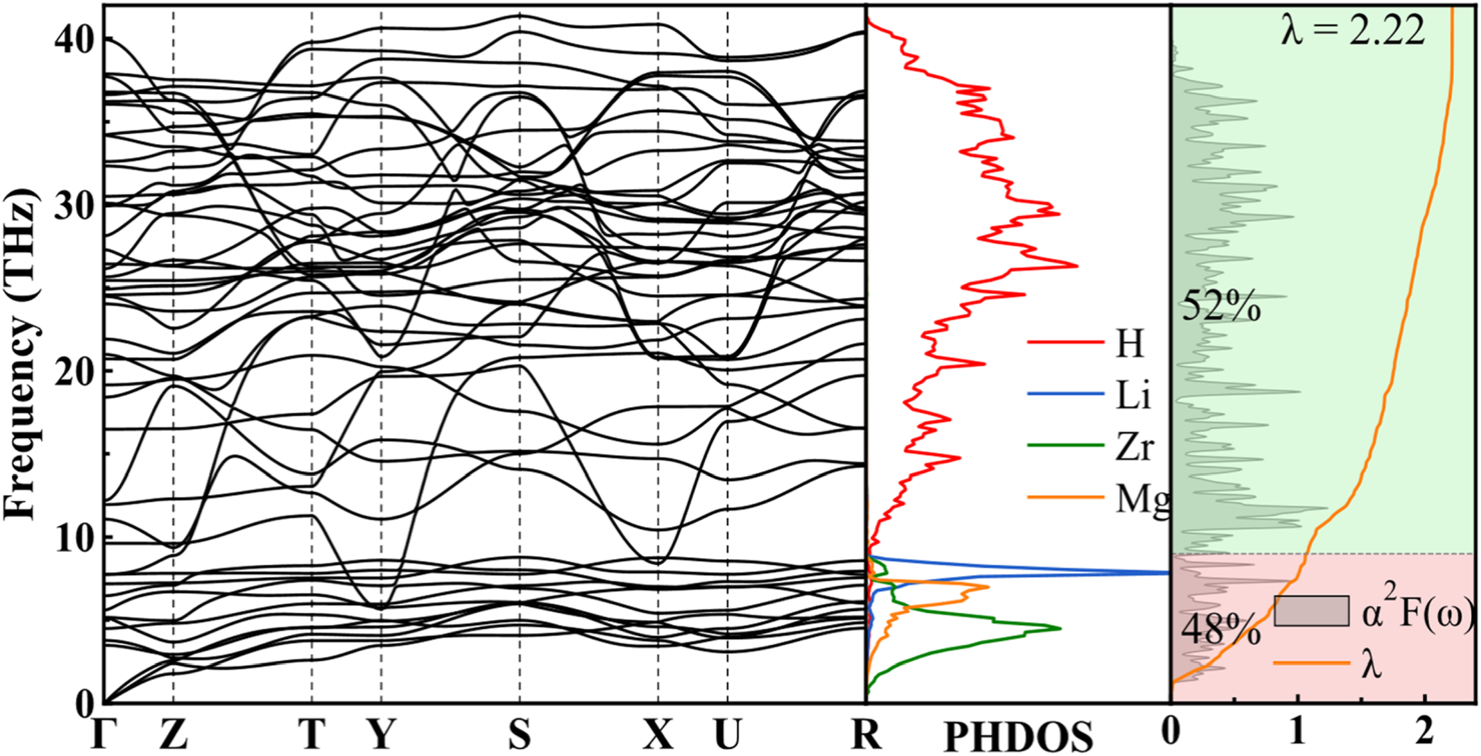
Calculated phonon dispersion curves, phonon density of states, Eliashberg phonon spectral function *α*^2^*F*(*ω*), and EPC intergraded *λ* for LiMgZr_2_H_12_.

Based on the MgZrH_6_ prototype, we constructed a LiMgZr_2_H_12_ structure with *Pmmm* symmetry and performed computational analyses of its thermodynamic, mechanical, and dynamical stability. The thermodynamic stability of the LiMgZr_2_H_12_ structure can be evaluated in terms of its formation energy, defined as follows:^28,29^1
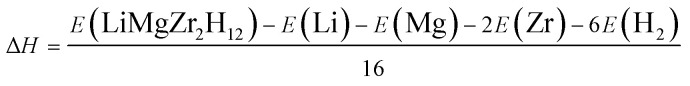
where *E*(LiMgZr_2_H_12_) denotes the total energy of the compound, *E*(H_2_) denotes the total energy of an H_2_ molecule, and *E*(Li), *E*(Mg) and *E*(Zr) denote the average energies of Li, Mg and Zr atoms in the crystals, respectively. The calculated formation energy of the LiMgZr_2_H_12_ structure is negative, indicating that the structure is thermodynamically stable with respect to decomposition into the pure elements. However, it is not necessarily thermodynamically stable against decomposition into other binary or ternary phases. Therefore, to further evaluate the thermodynamic stability of LiMgZr_2_H_12_, we systematically analyzed its possible decomposition pathways. Given the considerable challenge of constructing a complete phase diagram for a multicomponent compound, we assessed the thermodynamic stability of this structure only by comparing its relative formation enthalpy with respect to decomposition into the stable ternary, binary, and elemental phases identified in previous studies. As shown in Fig. 1(b), LiMgZr_2_H_12_ is thermodynamically unstable under ambient pressure and tends to decompose into other compounds, but it can be considered as a metastable phase. Such metastability does not preclude experimental synthesis, as approximately 20% of experimentally synthesized materials in the Inorganic Crystal Structure Database are metastable.^6,30–32^ For the quaternary hydride LiMgZr_2_H_12_, the *Pmmm* phase belongs to the orthorhombic crystal system. In this system, there are nine independent elastic constants: *C*_11_, *C*_12_, *C*_13_, *C*_22_, *C*_23_, *C*_33_, *C*_44_, *C*_55_, and *C*_66_. These values were calculated and listed in Table 1. The mechanical stability of the structure was assessed according to the Born stability criteria,^33^ which are given as follows: *C*_11_ > 0, *C*_22_ > 0, *C*_33_ > 0, *C*_44_ > 0, *C*_55_ > 0, *C*_66_ > 0, *C*_11_*C*_22_ > *C*_12_^2^, and 2*C*_12_*C*_13_*C*_23_ + *C*_11_*C*_22_*C*_33_ − *C*_11_*C*_23_^2^ − *C*_22_*C*_13_^2^ − *C*_33_*C*_12_^2^ > 0. The results show that LiMgZr_2_H_12_ satisfies the Born criteria, confirming its mechanical stability. Subsequently, we evaluated the dynamic stability of LiMgZr_2_H_12_ at ambient pressure by calculating its phonon dispersion. Fig. 2 shows that all phonon modes in the Brillouin zone exhibit positive frequencies, demonstrating that the LiMgZr_2_H_12_ structure is dynamically stable under ambient conditions.

**Table 1:** Calculation results of elastic constants *C*_*ij*_ (GPa) and formation energy Δ*H* (eV per atom) of LiMgZr_2_H_12_

	*C*_11_	*C*_12_	*C*_13_	*C*_22_	*C*_23_	*C*_33_	*C*_44_	*C*_55_	*C*_66_	Δ*H*
LiMgZr_2_H_12_	148.8	46.5	86.1	99.8	112.7	137.3	36.02	45.0	51.8	−0.128

Next, to assess the experimental feasibility of LiMgZr_2_H_12_, its potential synthesis route was explored. Inspired by previous studies, particularly the work of Yang *et al.*, we explored a possible synthesis route for LiMgZr_2_H_12_. Yang *et al.*^34^ synthesized a Mg–Zr–Li–H quaternary hydride with a *Fm*3̄*m* symmetry at 8 GPa and 873 K *via* the reaction 6MgH_2_ + ZrH_2_ + *n*LiH and found that the formation enthalpy of the quaternary phase is lower than that of Mg–Zr–H ternary hydrides. This result indicates that Mg–Zr–Li–H quaternary hydrides possess better thermodynamic stability. Inspired by the work of Yang *et al.*^4^,^34^ we propose a synthesis route for the LiMgZr_2_H_12_ quaternary hydride: LiMgZr_2_H_12_ → MgH_2_ + LiH + 2ZrH_2_ + 5/2H_2_. At ambient pressure, the calculated energy on the left side of the equation is only about 177 meV per atom higher than that on the right side, indicating that LiMgZr_2_H_12_ is a metastable phase. The reaction kinetics under ambient conditions may be slow for such solid metastable phases; thus, catalysts can be used to accelerate the reaction process. In addition, because metastable phases are prone to decomposition or phase transformation during synthesis, measures such as rapid quenching, sealing in quartz ampoules, or handling in an inert gas atmosphere are typically required.

The LiMgZr_2_H_12_ structure is composed of H_12_ cages centered by Zr, Li, and Mg atoms (Fig. 1(a)). Each H_12_ cage consists of 12 isosceles and 8 scalene triangles. LiMgZr_2_H_12_ exhibits a minimum H–H distance of 1.76 Å, which is substantially longer than the standard H–H covalent bond length in molecular H_2_ (0.74 Å) at ambient pressure, and slightly shorter than the shortest H–H distance in the parent MgZrH_6_ (1.82 Å). In addition, the average Zr–H bond length in LiMgZr_2_H_12_ is 2.09 Å and the average Mg–H bond length is 2.14 Å, both exceeding the corresponding Zr–H (1.97 Å) and Mg–H (1.99 Å) bond lengths in MgZrH_6_.^14^ These results indicate that, relative to the parent structure, substitutional Li doping modifies the local geometry of the hydrogen polyhedra, producing slight distortions of the H_12_ cages centered on Zr, Li, and Mg and yielding distinct coordination environments. Table 2 summarizes the detailed structural parameters of LiMgZr_2_H_12_ for further analysis.

**Table 2:** Structural parameters of LiMgZr_2_H_12_ at ambient pressure, including the crystal phase, lattice parameters (Å), Wyckoff positions, average Zr–H, Mg–H, and Li–H bond lengths (Å), and the shortest H–H distance (Å)

Phase	Lattice parameters	Wyckoff positions				H–H	Zr–H	Mg–H	Li–H
		Atoms	*x*	*y*	*z*				
*Pmmm*	*a* = 3.7833	Li (1f)	0.500	0.500	0	1.76	2.09	2.14	2.14
	*b* = 3.7853	Mg (1h)	0.500	0.500	0.500				
	*c* = 7.5389	Zr (2q)	0	0	0.245				
	*α* = *β* = *γ* = 90°	H1 (2r)	0	0.500	0.130				
		H2 (2r)	0	0.500	0.636				
		H3 (4v)	0.500	0.766	0.252				
		H4 (2i)	0.762	0	0				
		H5 (2j)	0.768	0	0.500				

To investigate potential superconductivity, we calculated the phonon dispersion relations, projected phonon density of states (PHDOS), Eliashberg spectral function *α*^2^*F*(*ω*), and the EPC constant *λ* for LiMgZr_2_H_12_ at ambient pressure. The corresponding results are summarized in Fig. 2. EPC analysis reveals that LiMgZr_2_H_12_ has an EPC constant *λ* = 2.22, which is significantly greater than that of MgB_2_ at ambient pressure (*λ* = 0.61)^35^ and that of MgZrH_6_ at 36 GPa (*λ* = 1.13),^14^ indicating the substantial EPC strength of LiMgZr_2_H_12_. Because of their relatively large atomic masses, Li, Zr, and Mg atoms are primarily associated with low-frequency phonon modes, whereas lighter H atoms dominate intermediate- and high-frequency phonon modes. In the low-frequency range (0–9 THz), the phonon modes primarily arise from the mixed vibrations of Li, Zr, and Mg atoms and contribute 48% to the total EPC constant *λ*. In contrast, the phonon modes in the intermediate- and high-frequency range (9–40 THz) are largely governed by H-atom vibrations, accounting for 52% of the total EPC constant *λ*. The *T*_c_ of LiMgZr_2_H_12_ was evaluated by solving the Allen–Dynes modified McMillan equation:^36,37^2
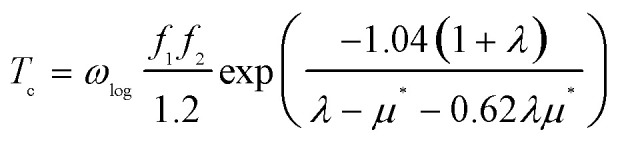
where *f*_1_ and *f*_2_ denote two correction factors, *ω*_log_ denotes the logarithmic average frequency, *λ* is the EPC constant, and µ* denotes the effective Coulomb repulsion. The definitions of *ω*_log_ and *λ* are given by:3
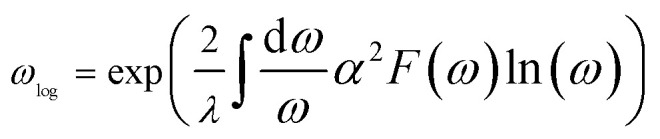
4
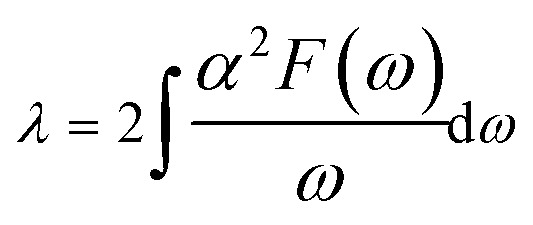
In our calculations, the Coulomb pseudopotential µ* was set to 0.10,^38–40^ and the corresponding results are summarized in Table 3. The calculations indicate that LiMgZr_2_H_12_ exhibits an estimated *T*_c_ of 60.8 K at ambient-pressure. Table 3 also lists the *T*_c_ of MgZrH_6_ at 36 GPa, as reported by Wang *et al.*,^14^ which was calculated using the Allen–Dynes modified McMillan equation. The table demonstrates that LiMgZr_2_H_12_ significantly reduces the external pressure required by the structure, while essentially maintaining the *T*_c_ of the parent structure. In addition, Table 3 also lists relevant data for several other typical ambient-pressure superconductors. The comparison shows that the *T*_c_ of LiMgZr_2_H_12_ is significantly higher than those of the ternary hydride superconductors YZrH_6_ (ref. 41) and Mg_2_IrH_6_,^43^ as well as the quaternary hydride superconductors MgCaIrH_6_,^44^ MgSrIrH_6_,^44^ and LiZrH_6_Ru,^45^ and is only slightly lower than that of YScH_6_,^42^ indicating that it exhibits relatively outstanding superconducting performance among ambient-pressure superconductors.

**Table 3:** EPC constant *λ*, logarithmic average phonon frequency *ω*_log_ (in K), density of states at the Fermi level *N*_F_ (in states per eV), *T*_c_ values (in K) estimated using the Allen–Dynes modified McMillan equation and superconducting figure of merit *S* for LiMgZr_2_H_12_ and other representative hydride superconductors reported in the literature under different pressures (in GPa)

Structures	Pressure	*λ*	*ω*_log_	*N*_F_	*T*_c_	*S*
LiMgZr_2_H_12_	0	2.22	396	2.34	60.8	1.56
MgZrH_6_ (ref. 14)	36	1.13		0.12	61.4	1.16
YZrH_6_ (ref. 41)	0	0.72	423		16.0	0.41
YScH_6_ (ref. 42)	0	1.31	598		66.5	1.71
Mg_2_IrH_6_ (ref. 43)	0	1.16	634		59.4	1.52
MgCaIrH_6_ (ref. 44)	0	1.54	285	1.28	33.4	0.86
MgSrIrH_6_ (ref. 44)	0	1.62	189	1.04	23.2	0.60
LiZrH_6_Ru^45^	0	1.00	342		23.5	0.60

To better evaluate the overall performance and practical applicability of the superconductor, we calculated the superconducting figure of merit *S*^46^ for LiMgZr_2_H_12_, which reflects the feasibility of a superconducting material for practical applications. The *S* parameter is defined as follows.5
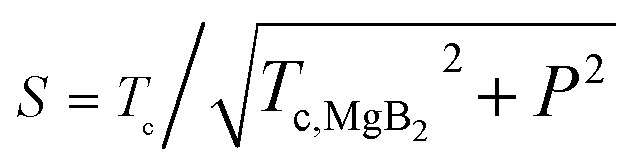
Here, *T*_c,MgB2_ denotes the superconducting critical temperature for MgB_2_, and *P* represents the applied pressure. The calculated *S* value of LiMgZr_2_H_12_ is 1.56, which is approximately 34% higher than that of the ternary hydrogen-rich compound MgZrH_6_. In addition, it outperforms a number of other representative, well-known superconducting materials. For example, the *S* values of experimentally synthesized H_3_S, YH_9_, and LaH_10_ have been evaluated as 1.27, 1.19 and 1.43, respectively;^1,3,4^ an *S* value of 1.23 has been reported for the recently synthesized LaBeH_8_ superconductor.^12^ This indicates that LiMgZr_2_H_12_ has outstanding potential for practical applications.

**Fig. 3:**
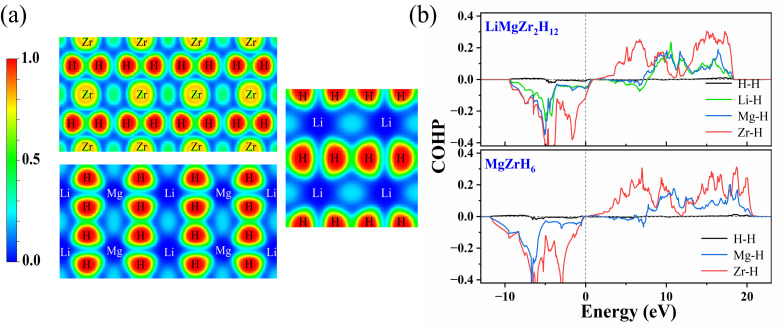
(a) Electronic localization function (ELF) of LiMgZr_2_H_12_. (b) The average calculated crystal orbital Hamiltonian populations (COHP) of selected atomic pairs in LiMgZr_2_H_12_ (0 GPa) and MgZrH_6_ (36 GPa).

The electron localization function (ELF) and Bader charges were also calculated, enabling the chemical bonding to be analysed. Fig. 3(a) shows the two-dimensional ELF map of LiMgZr_2_H_12_. The low ELF values between the metal and hydrogen atoms indicate ionic bonding, consistent with charge transfer from the metal atoms to hydrogen. The ELF value between the nearest neighbor H atoms is approximately 0.45, and no pronounced high-ELF shared regions are observed between H–H pairs, indicating the absence of significant covalent bonding between adjacent hydrogen atoms. A subsequent Bader charge analysis shows that each Li, Mg, and Zr atom donates approximately 0.86, 1.63, and 1.66*e*, respectively, while each H atom gains approximately 0.41–0.55*e*. This further confirms ionic interactions between the metal and hydrogen atoms, with hydrogen predominantly exhibiting hydride-like H^−^ character.

Subsequently, to evaluate the interactions between atoms, we calculated the COHP^47^ for selected atom pairs in LiMgZr_2_H_12_. As shown in Fig. 3(b), pronounced negative peaks appear below the Fermi level for the Zr–H, Mg–H, and Li–H bonds, indicating that bonding is primarily contributed by Zr–H, Mg–H, and Li–H interactions, which play a crucial role in stabilizing the structure. In contrast, the H–H COHP curve remains close to zero over the entire energy range and exhibits only minute oscillations, with almost no discernible peaks. This behavior suggests almost no interactions between neighboring H atoms, which is consistent with the results of the ELF analysis. Moreover, the deepest peak below the Fermi level is associated with the Zr–H bonds, indicating that Zr–H bonding is the strongest in this system. A comparison with the COHP curves of MgZrH_6_ reveals a key difference: in LiMgZr_2_H_12_, the Fermi level lies within a bonding region (negative COHP) dominated by Zr–H bonding states, whereas, in MgZrH_6_, the Fermi level is located near the boundary between bonding and antibonding states, where the COHP is close to zero. These results indicate that the introduction of Li atoms shifts the Fermi level into a pronounced bonding region, significantly enhancing the electronic density of states at the Fermi level.

**Fig. 4:**
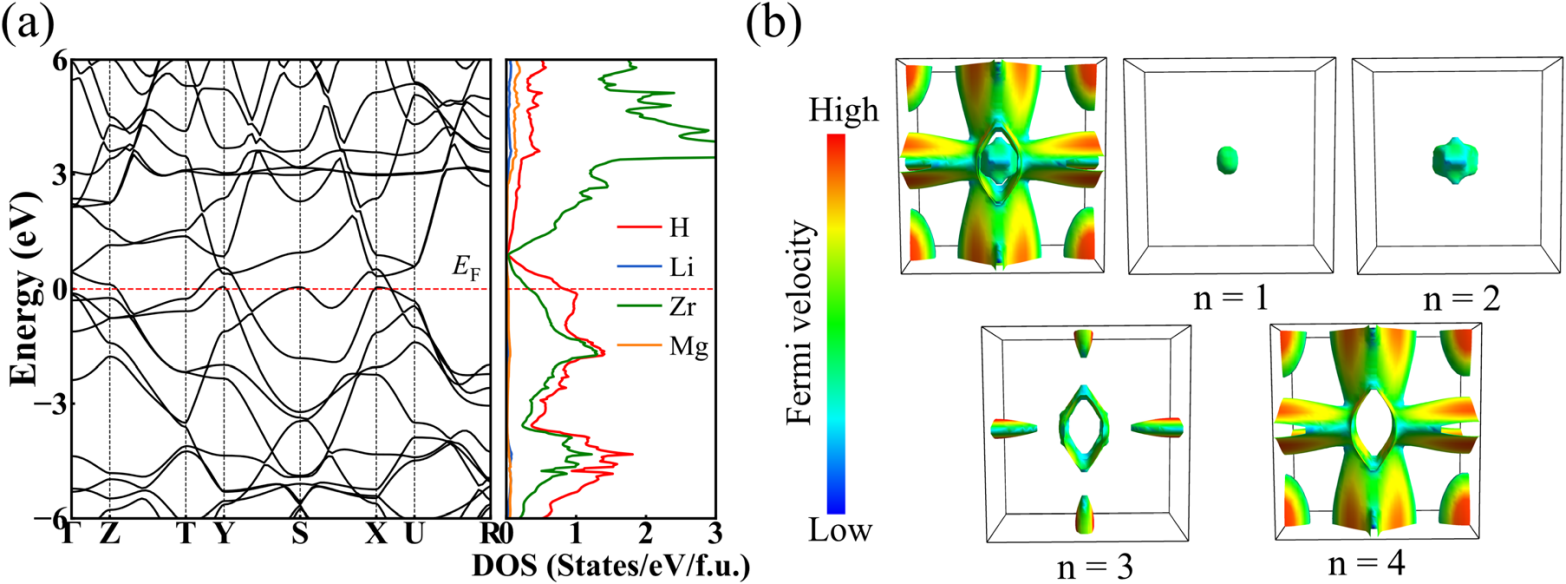
(a) Calculated band structures and PDOS for LiMgZr_2_H_12_ under ambient pressure. The dashed line at zero indicates the Fermi energy. (b) Fermi surface topology of LiMgZr_2_H_12_.

The electronic structure of a material is closely related to its superconductivity. Thus, we further investigated the band structures and the projected density of states (PDOS) of LiMgZr_2_H_12_, as shown in Fig. 4(a). The band structure shows several steep bands cross the Fermi level, indicating metallic behavior. The PDOS plot further shows that the electronic states near the Fermi level originate mainly from Zr and H. Combined with the preceding COHP analysis, which indicates that H–H interactions are essentially nonbonding across the entire energy range, this implies that the hydrogen contribution near the Fermi level stems predominantly from Zr–H hybridized states rather than H–H metallic bonding. In addition, distinct van Hove singularities appear near the Fermi level at the Y, S, and X points, which can effectively enhance superconductivity. The overall density of states profile of LiMgZr_2_H_12_ is similar to that of *Pm*3̄ -MgZrH_6_ at 36 GPa.^14^ However, due to the relative downward shift of the Fermi level in LiMgZr_2_H_12_, the contribution of H and Zr atoms to the density of states near the Fermi level significantly increases, thereby enhancing the total density of states (TDOS) near the Fermi level. In addition, the density of states near the Fermi level in the LiMgZr_2_H_12_ structure is primarily dominated by hydrogen atoms. The contribution of hydrogen near the Fermi level to the TDOS is significantly higher than those of the three metal elements Li, Zr, and Mg. This high hydrogen-derived DOS may enable more electrons to participate in Cooper-pair formation. According to BCS theory,^48^ a larger TDOS and H-dominated electronic states at the Fermi level generally favor stronger EPC and a higher *T*_c_. This explains why LiMgZr_2_H_12_ exhibits superior superconducting properties compared to MgZrH_6_. Notably, two bands with almost parallel dispersion are found to intersect the Fermi level along the T–Y direction. The presence of such nearly parallel dispersive features in the band structure indicates potential Fermi surface nesting, whose strength is largely governed by the geometry of the Fermi sheets. The Fermi surface topology of LiMgZr_2_H_12_ in the Brillouin zone was calculated. As shown in Fig. 4(b), four conduction bands cross the Fermi level in LiMgZr_2_H_12_. Here, *n* denotes the band index. For *n* = 1 and 2, the corresponding Fermi surfaces form closed, smooth, nearly spherical pockets, indicating typical metallic behavior. The *n* = 3 band generates a rhombic closed “inner-shell” Fermi surface, whose four sides contain extended, nearly flat facets that are approximately parallel to the Fermi sheets formed by the *n* = 4 band, resulting in interband nesting channels. Importantly, such nesting may induce phonon softening or Kohn anomalies and enhance EPC, thereby playing a crucial role in strengthening superconductivity.^49^

## 4. Discussion

Compared with the parent *Pm*3̄ -MgZrH_6_,^14^ the LiMgZr_2_H_12_ structure obtained through Li substitution exhibits a markedly enhanced hydrogen-derived contribution near the Fermi level, while the contributions from Li and Mg near the Fermi level remain weak. This indicates that LiMgZr_2_H_12_ does not strengthen superconductivity by directly introducing Li-related metallic states, but rather by restructuring the Zr–H framework and increasing the hydrogen weight in Zr–H hybridized states near the Fermi level. Structurally, substitutional Li doping induces distortions of the hydrogen-containing polyhedra. This polyhedral reconstruction and the resulting changes in coordination environments modify the local force constants and the vibrational characteristics of the relevant atoms. According to lattice-dynamics theory, such changes in force constants alter phonon frequencies and thereby affect the EPC strength.^50,51^ EPC analysis further indicates that approximately 52% of the EPC in LiMgZr_2_H_12_ originates from the high-frequency region, which is predominantly governed by hydrogen vibrations. By contrast, the EPC in the parent MgZrH_6_ depends more on low-frequency contributions. Therefore, Li doping not only increases the hydrogen contribution to the TDOS near the Fermi level, but also enhances the electron–phonon interaction between electronic states near the Fermi level and H-dominated vibrational modes, leading to a marked increase in the electron–phonon coupling constant *λ* and consequently improving the superconducting performance.

## 5. Conclusion

In summary, inspired by recent studies on the MgZrH_2*n*_ series, we constructed a LiMgZr_2_H_12_ structure with *Pmmm* symmetry and investigated its stability, electronic properties, and superconductivity using first-principles calculations. The ELF results indicate that there is almost no interaction between H–H pairs in LiMgZr_2_H_12_ and that the interactions between the metal and hydrogen atoms are predominantly ionic. Furthermore, COHP analysis shows that the Zr–H interaction is the strongest and plays a crucial role in stabilizing the structure. EPC analysis demonstrates that LiMgZr_2_H_12_ remains dynamically stable at ambient pressure and exhibits a high *T*_c_ of 60.8 K. Compared with MgZrH_6_, LiMgZr_2_H_12_ significantly reduces the external pressure required by the structure while essentially maintaining the *T*_c_ of the parent structure. This improvement can be attributed to the high electronic DOS at the Fermi level and the strong EPC in LiMgZr_2_H_12_. H-dominated electronic states at the Fermi level is another key factor that enhances its superconducting performance. Moreover, the calculated superconducting figure of merit *S* of LiMgZr_2_H_12_ is 1.56, which is approximately 34% greater than that of MgZrH_6_ in the ternary system, indicating substantial potential for practical applications. Our study provides theoretical guidance for future experimental work and offers valuable insights into the exploration of quaternary superconducting hydrides, which remain largely unexplored to date.

## Author contributions

Qun Wei: Supervision, project administration, writing – review & editing. Xinyu Wang: investigation, data curation, writing – original draft. Jing Luo: data curation, investigation. Meiguang Zhang: writing–review & editing, resources, funding acquisition. Bing Wei: writing–review & editing, resources, supervision.

## Conflicts of interest

There are no conflicts to declare.

## Data Availability

All data used in the analysis can be found within this manuscript.
